# Curing and Characteristics of N,N,N′,N′-Tetraepoxypropyl-4,4′-Diaminodiphenylmethane Epoxy Resin-Based Buoyancy Material

**DOI:** 10.3390/polym11071137

**Published:** 2019-07-03

**Authors:** Sizhu Yu, Xiaodong Li, Xiaoyan Guo, Zhiren Li, Meishuai Zou

**Affiliations:** School of Materials Science and Engineering, Beijing Institute of Technology, Beijing 100081, China

**Keywords:** buoyancy material, epoxy resin, DSC, thermal analysis kinetics

## Abstract

Buoyancy material is a type of low-density and high-strength composite material which can provide sufficient buoyancy with deep submersibles. A new buoyancy material with N,N,N′,N′-tetraepoxypropyl-4,4′-diaminodiphenylmethane epoxy resin (AG-80) and m-xylylenediamine (m-XDA) curing agent as matrix and hollow glass microsphere (HGM) as the filler is prepared. The temperature and time of the curing process were determined by the calculations of thermal analysis kinetics (TAK) through differential scanning calorimetry (DSC) analysis. The results show that the better mass ratio of AG-80 with m-XDA is 100/26. Combined TAK calculations and experimental results lead to the following curing process: pre-curing at 75 °C for 2 h, curing at 90 °C for 2 h, and post-curing at 100 °C for 2 h. The bulk density, compressive strength, and saturated water absorption of AG-80 epoxy resin-based buoyancy material were 0.729 g/cm^3^, 108.78 MPa, and 1.23%, respectively. Moreover, this type of buoyancy material can resist the temperature of 250 °C.

## 1. Introduction

As the detection of the oceans by humans deepens, buoyancy materials that provide net buoyancy to deep-sea probes are increasingly needed. High-strength buoyancy materials are widely used in civil, commercial, and military applications, such as counterweights in water equipment, zero buoyancy tows, and unmanned remote diving. Buoyancy material is essentially a type of low-density, high-strength, and low-water-absorbing composite material, which commonly consists of resin matrix and filler material [1,2,3,4,5,6,7]. Since epoxy resin has excellent mechanical properties, high adhesiveness to many substrates, and low water absorption, currently it is intensively used as the resin matrix of buoyancy materials [8,9,10,11]. Epoxy resin is defined as low-molecular-weight pre-polymers with two or more epoxy groups [12]. With different combinations of epoxy resins and curing agents, the properties become different [13,14,15,16,17]. N,N,N′,N′-tetraepoxypropyl-4,4′-diaminodiphenylmethane (AG-80) epoxy resin is a type of diglycidylamine epoxy resin which is characterized by low viscosity, high activity, low shrinkage, and excellent mechanical properties [18,19]. Compared with bisphenol A type epoxy resin, the most widely used in the market, the activity of AG-80 epoxy resin is found to be 10 times, and the tertiary amine of AG-80 promotes the ring-opening of the epoxy group [20,21].

During the curing process of epoxy resin, many different chemical reactions occur simultaneously [22,23]. To simplify, thermal analysis kinetics (TAK) method is used to simulate the curing process of epoxy resin [24,25,26,27], and the curing process is determined by the TAK calculations and experimental results. TAK is a method that quantitatively characterizes the chemical reaction process or physical changes of the substance by the technique of thermal analysis. Through mathematical processing, the kinetic parameters such as activation energy (*E*), pre-exponential factor (*A*), and reaction rate constant (*k*) are obtained to evaluate the stability [28], lifetime [29], and generation process [30] of epoxy resin. The traditional TAK method is isothermal method, along with the technical development, the nonisothermal method appears. As the nonisothermal method is rapid, simple, and easy to implement, it has gradually become the primary way of TAK method [31]. Among the various methods in thermal analysis kinetics, the Flynn–Wall–Ozawa (FWO) method is an equal conversion rate method, which is analyzed by processing the data points at the same conversion rate on a kinetic curve at different heating rates. Furthermore, the kinetic parameters are obtained without calculating the kinetic mode function, so that FWO method is one of the non-model methods, which simplifies the calculation process.

In this paper, the curing process between AG-80 epoxy resin and m-xylylenediamine (m-XDA) curing agent was studied. TAK method was used to calculate the apparent activation energy (*E_a_*), which established the curing process of AG-80 epoxy resin. Hollow glass microsphere (HGM) with different volume fractions (*V*_H_) was added into the AG-80 epoxy resin matrix to produce materials with low-density, high-strength, and low saturated water absorption properties, which have potential applications as deep-sea buoyancy materials.

## 2. Materials and Methods

### 2.1. Materials and Experimental Procedure

N,N,N′,N′-tetraepoxypropyl-4,4′-diaminodiphenylmethane (AG-80) epoxy resin with an epoxy value of 0.75–0.85 was obtained from Shanghai Institute of Synthetic Resins, Shanghai, China. m-xylylenediamine (m-XDA) of 99% was received from Shanghai Macklin Biochemical Co., Ltd., Shanghai, China and hollow glass microspheres (HGM) of H32 type were procured from Sinosteel Maanshan Mine New Material Technology Co., Ltd., Anhui, China.

A specific amount of AG-80 epoxy resin was weighed and placed in a vacuum drying oven at 80 °C for 30 min, and then cooled at room temperature. Then, m-XDA was weighed at different calculated ratio (the mass ratio of AG-80 epoxy resin and m-XDA was 100/20, 100/22, 100/24, 100/26, 100/28, 100/30, respectively) and added to the cooled epoxy resin, then well mixed at the stirring speed of 50 rpm. The mixtures at different ratio were used to test the curing kinetics by differential scanning calorimetry (DSC) testing. For the preparation of AG-80 epoxy resin-based buoyancy materials, the mixture (the mass ratio of AG-80 epoxy resin and m-XDA was 100/26) was prepared for the same procedure as above. The HGM which was dried at 80 °C for 2 h was added to AG-80/m-XDA system slowly at different volume fraction (0%, 40%, 45%, 50%, 55%, respectively) and mixed at a stirring speed of 10 rpm. After uniform mixing, the mixed system was poured into a mold and placed in a vacuum drying oven at room temperature for 50 min to eliminate the bubbles. Then, took them out at the curing conditions, which were determined by TAK calculations as well as experimental results (pre-curing at 75 °C for 2 h, curing at 90 °C for 2 h, and post-curing at 100 °C for 2 h).

### 2.2. Differential Scanning Calorimeter (DSC)

All the nonisothermal tests related to AG-80/m-XDA system (at different mass ratio and heating rates) were carried on a differential scanning calorimeter (DSC, 204 F1, (Netzsch Co. Ltd., Freistaat, Germany) with an intercooler (IC40) calibrated with the standard. The parameters for the DSC test were: mass of samples at 5~8 mg, aluminum crucibles, nitrogen atmosphere, sweeping rate was 20 mL/min, shielding gas rate was 60 mL/min, and the temperature range was 30~180 °C. AG-80/m-XDA at different mass ratios (100/20, 100/22, 100/24, 100/26, 100/28, 100/30, respectively) were used at the heating rate of 2.5 K/min. Following this, specific ratio (100/26) of AG-80/m-XDA varying rates of heating, i.e., 2.5 K/min, 5 K/min, 10 K/min, 15 K/min, and 20 K/min, were applied.

### 2.3. Fourier Transform Infrared Spectroscopy (FTIR)

Uncured liquid AG-80 and the samples cured with m-XDA under the optimized conditions (air background, the number of scans is 32 times, and the wavenumber range from 4000 cm^−1^ to 400 cm^−1^) were tested by infrared spectrometer (FTIR, 6700, Nicolet, Madison, WI, USA).

### 2.4. Mechanical Performance

The uniaxial compression strength of AG-80 epoxy resin-based buoyancy materials was tested according to the standard of test methods for properties of resin casting boby (GB/T2567-2008). The cylindrical samples of Φ10 mm × 25 mm were tested at a compression rate of 2 mm/min by electronic universal material testing machine (5985, INSTRON Corporation, Boston, MA, USA). The 5 samples were subjected to testing, and the average of these was taken as the final result.

### 2.5. Saturated Water Absorption

The samples were processed into square pieces of 50 mm × 50 mm × 2 mm and dried in a forced air oven at 50 °C for 24 h, and then cooled to room temperature. The samples were weighed (*m*_1_) and completely immersed in deionized water at room temperature. The final weight of the sample (*m*_n_) was used to calculate the percentage of saturated water absorption.

### 2.6. Scanning Electron Microscopy (SEM)

To observe the fractured surface of the solid buoyancy materials, SEM was employed (TM3000 desktop electronic scanning mirror, Hitachi, Tokyo, Japan).

### 2.7. Thermogravimetric Analysis (TGA)

The thermal decomposition of AG-80/m-XDA system (the mass ratio is 100/26) and AG-80 epoxy resin-based buoyancy material (*V*_H_ = 55%) were examined by a thermogravimetric analyzer (TGA/DSC3+/668, METTLER TOLEDO, Zurich, Switzerland) with the heating rate of 10 °C/min from 30 °C to 800 °C under the N_2_ protection (50 mL/min). About 5 mg of the samples were charged in the alumina crucible without a lid.

## 3. Results and Discussion

### 3.1. Nonisothermal Curing of Epoxy Resin

The curing agent used in this study is a type of aromatic amine and the theoretical amount range of which could be calculated by Equation (1):

(1) $$phr=\frac{M}{n}\times e$$

where *phr* is the amount of curing agent and represents the mass fraction of curing agent required per 100 parts of the resin, *M* is the molecular weight of the amine, i.e., m-XDA, *n* is the number of active hydrogen present in the amine, and *e* is the epoxy value. The *phr* of m-XDA was calculated to be 25.53~28.94.

**Figure 1:**
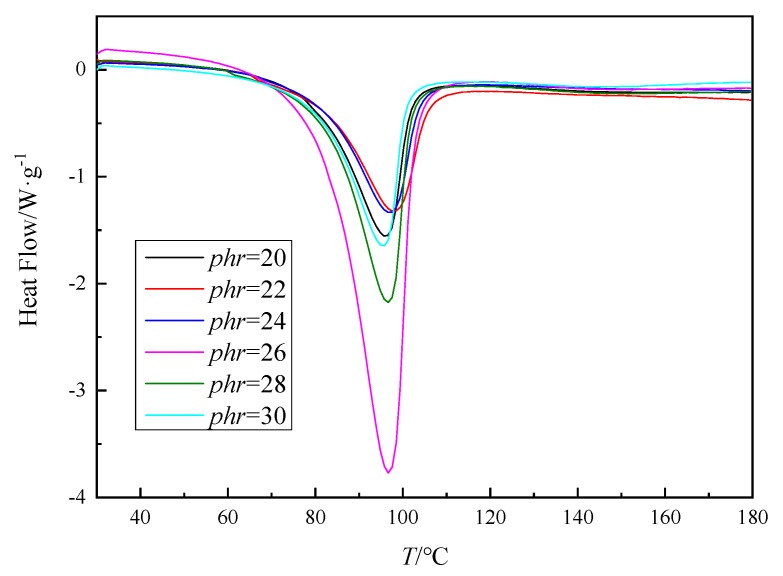
The differential scanning calorimetry (DSC) curves for N,N,N′,N′-tetraepoxypropyl-4,4′-diaminodiphenylmethane epoxy resin (AG-80)/m-xylylenediamine (m-XDA) curing system with different mass ratios (*phr* = 20, 22, 24, 26, 28, 30) at a rate of 2.5 K/min.

Based on Equation (1), the range for the theoretical mass ratio of AG-80 resin and m-XDA can be calculated. However, the optimal ratio still needs to be screened by the exothermic of nonisothermal DSC curves. The mass of m-XDA was increased by a gradient in the calculated ratio, where *phr* = 20, 22, 24, 26, 28, and 30. In case of actual industrial production, three-stage constant temperature curing process of pre-curing, curing, and post-curing are generally adopted [32], and the curing is relatively close to a constant temperature at a heating rate of 2.5 K/min. Figure 1 shows the DSC curves for different ratios of AG-80/m-XDA at 2.5 K/min. All the DSC curves show only a single exothermic peak, which indicates that the only reaction involved is the amine-epoxy addition without interference of side reactions. Table 1 shows the exothermic values of the curing system. It could be noted that AG-80/m-XDA system has the most substantial amount of heat release when *phr* = 26. This could be due to that when the amount of curing agent is low, the reaction is not entirely due to insufficient content of active hydrogen, i.e., less amount of heat is released (*phr* = 20, 22, and 24, as shown in Table 1). When the addition of curing agent is higher than stoichiometric value, the decreasing of evolved heat is probably due to a lower amount of epoxy (per gram of sample), therefore, some NH_2_ and NH groups cannot react because of lack of epoxies [33]. From that above, it has been confirmed that the optimum ratio of AG-80/m-XDA curing system was *phr* = 26.

**Figure 2:**
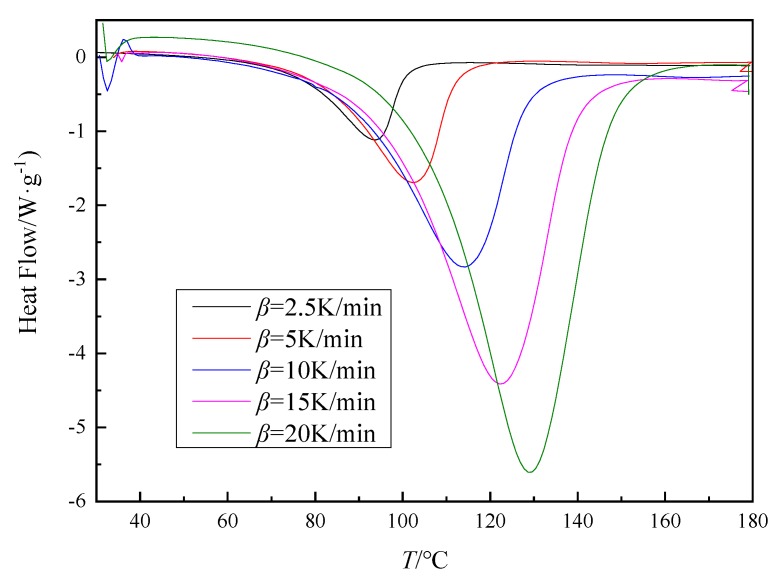
The DSC curves for AG-80/m-XDA curing system (*phr* = 26) with different heating rates (*β* = 2.5 K/min, 5 K/min, 10 K/min, 15 K/min, and 20 K/min).

For AG-80 epoxy resin and m-XDA mixed at an optimum ratio (*phr* = 26), DSC test was used at different heating rates (2.5 K/min, 5 K/min, 10 K/min, 15 K/min, and 20 K/min). Figure 2 shows the AG-80/m-XDA curing system (*phr* = 26) at different heating rates (*β* = 2.5 K/min, 5 K/min, 10 K/min, 15 K/min, and 20 K/min). All the DSC curves show only a single exothermic peak, which indicates that the only reaction involved is the amine-epoxy addition without interference of side reactions. As shown in Figure 2, the initial temperature *T*_i_, the peak temperature *T*_p_, and the final temperature *T*_f_ of the curing reaction were obtained by DSC curves, and the values are shown in Table 2. It can be seen from Figure 2 and Table 2 that the exothermic peak is continuously steepened as the heating rate increases and the isothermal reaction enthalpy changed, indicating that the degree of cure is different as the heating rate increases. At the same time, with the increase of the heating rate, the exothermic peak of curing reaction and characteristic temperatures (*T*_i_, *T*_p_, and *T*_f_) of the system move toward high temperature. That is because the thermal inertia becomes higher with the heating rates increasing, the heat released by AG-80/m-XDA system per unit time is higher, which causes the temperature difference is broader. Thus, the curing exothermic peaks and the characteristic temperatures move to the high-temperature direction.

In reality, curing reactions of epoxy resins are rather complex, complicated by plenty of elementary reactions, mass-transfer process, and phase changes, which will result in rather complex reaction mechanisms [34]. To gain a deeper insight into the isothermal reaction of AG-80/m-XDA system, here the classic method (Flynn–Wall–Ozawa method) is used to analyze the curing kinetics. The Flynn–Wall–Ozawa (FWO) method [35,36] is shown as following:

(2) $$\mathrm{lg}\beta =\mathrm{lg}\left[\frac{AE_{a}}{RG(\alpha )}\right]-2.315-0.4567\frac{E_{a}}{RT}$$

where *β* is the heating rate, R is the universal gas constant, *E_a_* is the apparent activation energy, *A* is the pre-exponential factor, *α* is the conversion rate, and *G(α*) is the integral form of the reaction mechanism function.

**Figure 3:**
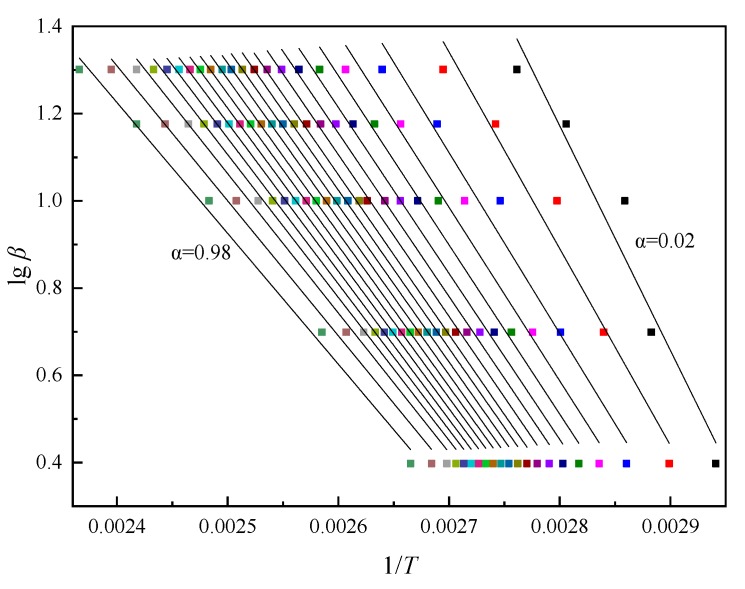
The lg*β*-1/*T* fitting curves of AG-80/m-XDA system (*phr* = 26) at different conversion rates (*α* = 0.02~0.98) by Flynn–Wall–Ozawa (FWO) method.

According to Equation (2), the temperature corresponding to each conversion rate of varying *β* could be found from the curves in Figure 2. Figure 3 shows the lg*β*-1/*T* fitting curves of AG-80/m-XDA system (*phr* = 26) at different conversion rates (*α*) according to FWO method. For the curing system, the slope of the regression curve at each conversion rate is used to obtain *E_a_* at the corresponding conversion rate, and the results are shown in Table 3. As illustrated in Table 3, *E_a_* depends on *α*, which suggests the reaction seems to follow the multi-step reaction mechanisms associated with the different kinetic steps with varying energetic barriers [37]. Table 3 shows that for each curing system, with an increase of *α*, the *E_a_* of the system tends to decrease, which could mirror the drastic change of the reaction mechanisms, in particular, the drop in *E_a_* observed in the deep-conversion rate regime due to the diffusion-controlled reaction kinetics [38].

**Figure 4:**
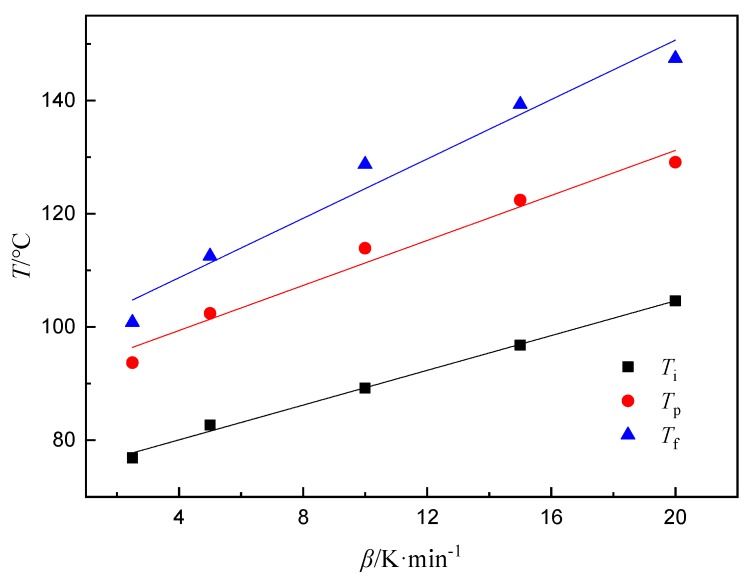
The fitting curves of characteristic temperatures for AG-80/m-XDA curing system (*phr* = 26) with different heating rates.

The curing process of epoxy resin in industrial production generally adopts a staged constant temperature curing method, and the curing temperature of each stage is closely related to *T*_i_, *T*_p_, and *T*_f_ [39]. The curing process temperature is usually determined by extrapolation, that is, the temperature at which the curing reaction occurs is linearly related to the heating rate [40]. By extrapolating the *T*_i_, the gel temperature is obtained; by extrapolating the *T*_p_, the curing temperature is obtained; and by extrapolating the *T*_f_, the posttreatment temperature is obtained, which are the process parameters of the curing reaction system. The curing process consists of curing time and temperature which were obtained by *E_a_* (Table 3) at different α and characteristic temperatures (Table 2), respectively. Through the fitting curve of *T*-*β* (Figure 4), the temperature extrapolated for *β* = 0 is the curing temperature (Table 4).

The time when the theoretical degree of curing system reaches 100% can be calculated by the pre-exponential factor *A* and the reaction order *n*. However, n-level mode equation cannot calculate the correct pre-exponential factor *A*, so this method is not available. The non-model method which uses different apparent activation energy with different degree of cure to calculate the relationship between the curing time and the degree of solidification at the isothermal curing temperature does not need to calculate the pre-exponential factor *A*. Thus, non-model method [41] is in good agreement with the actual results. The non-model method is shown in Equation (3).

(3) $$t_{\alpha }=\frac{\int_{0}^{T_{\alpha }}\exp\left[-E_{\left(\alpha \right)}/\left(RT\right)\right]dT}{\beta exp\left[-E_{\left(\alpha \right)}/\left(RT_{iso}\right)\right]}$$

where *T_α_* is the temperature when the conversion rate is *α*, *T*_iso_ is the isothermal curing temperature, *T* is the curing temperature at different heating rates, and *E_(α)_* is the apparent activation energy for a specific conversion rate *α*.

**Figure 5:**
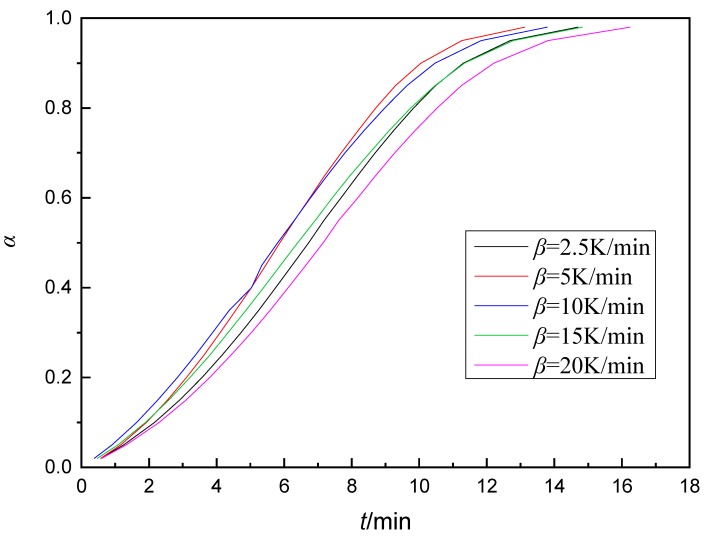
The conversion-time curves of AG-80/m-XDA system (*phr* = 26) calculated by non-model method at 90 °C with different heating rates (*β* = 2.5 K/min, 5 K/min, 10 K/min, 15 K/min, and 20 K/min).

Figure 5 shows the *α*-*t* curves of AG-80/m-XDA system, which was calculated by Equation (3) and curing temperature of 90 °C was chosen for the calculations. By comparing the five different heating rates, it could be noted that the five curves are consistent within the limits of the required error tolerance. This illustrates the accuracy of the fitting data. In Figure 5, the curves can be divided into three stages which correspond to two changes in the structure of the epoxy curing process-gel reaction and glass transform. With an increase in curing time, the degree of curing at first increase slowly, then rise quickly and then again increased gradually, which could be related to the changes of *E_a_*. In Figure 5, the curing degree reaches about 80% during 10 min, but the glass transform affects the system as the curing progresses apparently. Therefore, the time required for the degree of curing to reach 100% is much longer than 10 min. Considering the above calculation results and the final curing process of the experiments, the curing process of AG-80/m-XDA system is 75 °C for 2 h, 90 °C for 2 h, and 100 °C for 2 h.

### 3.2. The Comprehensive Performance of Buoyancy Material

**Figure 6:**
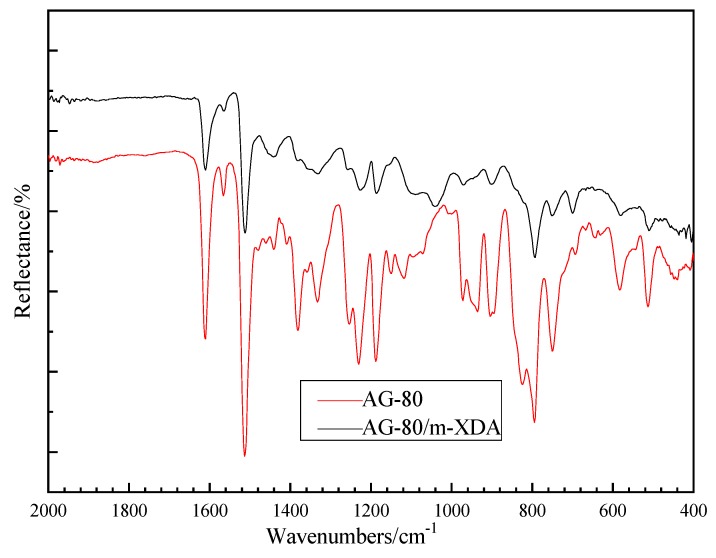
The FTIR curves of AG-80 epoxy resin and AG-80/m-XDA cured system (*phr* = 26).

The degree of curing reaction can be estimated by the comparison of the group contents with FTIR curves. Figure 6 shows the FTIR spectrum curves of AG-80 and AG-80/m-XDA cured system (*phr* = 26), and it can be seen that the characteristic peak of the epoxy group near 910 cm^−1^ is significantly reduced after curing. Hydrogen attacks the epoxy group of AG-80 and opens the epoxy bond to form a hydroxyl group, and the amino group that lost hydrogen bonded to the methylene group forms a secondary amino group, and then the hydrogen of the secondary amino group attacks the epoxy group and forms a tertiary amino group. With the formation of a crosslinked network structure, the content of epoxy groups is significantly reduced.

The current requirements for buoyancy materials are lower density and higher strength to provide more net buoyancy and withstand higher pressures. Thus, density and compressive strength are the main criteria for testing the buoyancy materials. Table 5 shows the comprehensive properties of cured AG-80 epoxy resin (*phr* = 26) matrix and the AG-80 epoxy resin-based buoyancy materials with different volume fractions of HGM. For the density of buoyancy material, the theoretical density could be calculated (the density of the AG-80/m-XDA system is 1.208 g/cm^3^; the average density of the H32 type HGM is 0.32 g/cm^3^), and the actual density was measured by the drainage method, and the specific values are shown in Table 5. The results of density show that as the content of HGM in the buoyancy material increases, the density of buoyancy materials decrease. Moreover, the actual density of the material is always near the calculated density, that is, the sample preparation tends to be perfect. The actual density of sample No. 2 is less than the calculated density. The reason could be that the viscosity of the system is significant during the preparation of the sample, and a small number of air bubbles are mixed during mixing. During the process, the bubbles are not completely removed as the actual density of the sample is less than the ideal density due to the presence of air bubbles. With the increase of HGM *V*_H_, the actual density of samples No. 3–5 is higher than the theoretical density. At this time, the *V*_H_ of HGM is more significant than or equal to 45%. The reason could be that during the preparation of the sample, as the HGM content continues to increase, the viscosity of the system becomes more substantial. During the process of mixing and stirring, the thinner HGM of the shell wall breaks, which causes that the air inside the HGM is substituted by the epoxy resin matrix. Thus, the actual density of the system was higher than the value of theoretical calculation.

Since the basic requirements of buoyancy materials are high strength and low density, the intensity-to-density ratio (*E/ρ*) can be used to describe the quality of the prepared samples. Table 5 shows the relationship between *E/ρ* and HGM *V*_H_ of samples. It can be seen from Table 5 that as the *V*_H_ of HGM increases, the value of sample *E/ρ* becomes higher. When the *V*_H_ increased to 55%, the viscosity of the sample preparation system became high, resulting into an inability to prepare a sample in which the distribution of HGM is uniform, and no bubbles were present. The fluidity of HGM is not very good, and the viscosity is affected when the added *V*_H_ is significant if more HGM is added. Thus, without thinner adding, the ultimate volume fraction of HGM for this system is 55%.

The filler of buoyancy material is HGM which is a type of inorganic material, and the matrix of buoyancy material is AG-80 epoxy resin which is a type of organic material, so it has a problem of poor adhesion between the two phases. The compressive strength of the prepared samples is affected by this factor. Combining the effects of various factors, the empirical model [42] of the compressive strength of the composite is established (Equation (4)).

(4) $$\sigma_{c}=\sigma_{m}\frac{1-V_{H}}{1+\delta V_{H}}\exp\left(\varepsilon V_{H}\right)$$

where *σ*_c_ is the compressive strength of the composite; *σ*_m_ is the strength of the resin matrix; *V*_H_ is the volume fraction of HGM; *δ* is the filler form factor; HGM is spherical, *δ* is 2.5; and *ε* is the interface bonding and properties of HGM. When *ε* = 0, the system has no bonding and when *ε* ≥ 3, the system is well bonded.

**Figure 7:**
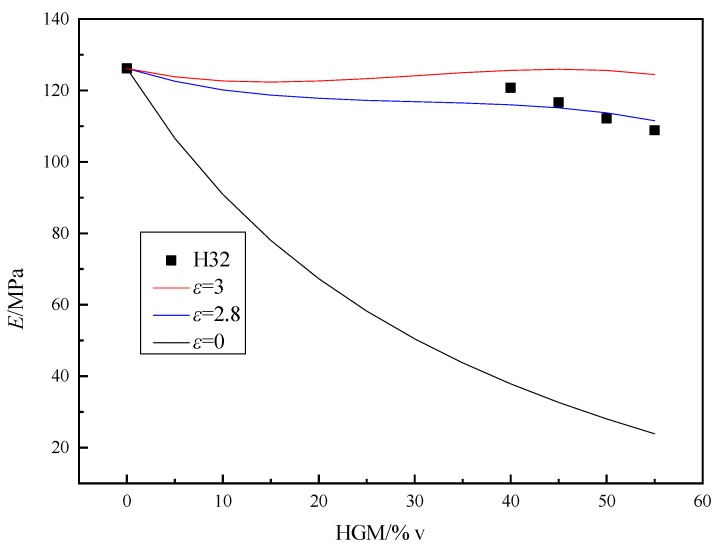
The relationship between the compressive strength and volume fraction of AG-80 epoxy resin-based buoyancy materials.

Table 5 shows the compressive strength of the sample prepared by using H32-type HGM and the calculated strength is obtained by Equation (4). In Figure 7, *ε* = 0 and *ε* = 3 are the curves calculated by Equation (4), and the fitting curve of *ε* = 2.8 is the optimal one obtained by substituting the intensity of the sample into Equation (4). As can be seen from Figure 7 that when *ε* = 2.8, the compressive strength of the sample as a function of *V*_H_ can be better described. The physical meaning of the curve of *ε* = 0 in Figure 7 is the trend of changes in the compressive strength of the ordinary foam material. As *ε* becomes higher, the bonding condition of the system gradually becomes better, and the obtained *ε* = 2.8 shows that the epoxy resin system and HGM bonding are better.

The compressive strength of the prepared samples is also affected by other factors, such as the bubble content of the samples. Figure 7 shows the relationship between compressive strength and the *V*_H_ of the samples. It could be seen from Figure 7 that as the *V*_H_ of HGM increases, the compressive strength of the sample decreased, and the degree of decrease for compressive strength is also increased. This is because the compressive strength of HGM is much lower than matrix′s. When the HGM content is low, it is mainly matrix resin. The shear failure causes the sample to break. With an increase for the content of HGM, the failure mode become the comprehensive failure mode of the shear failure of the matrix resin and the collapse failure caused by HGM crushing. The collapse of HGM is the primary manifestation. For samples prepared using H32 type of HGM, the reduction in compressive strength is not substantial, probably due to that the H32-type HGM itself has a higher strength.

The gap between the HGM and the epoxy resin matrix is the key factor of increasing the water absorption rate. The saturated water absorption rate (*c_s_*) is calculated using the following Equation (5).

(5) $$c_{s}=\frac{m_{n}-m_{1}}{m_{1}}$$

As above, the No. 5 sample has the best properties and hence is chosen to test the water absorption and SEM. The relationship between the water absorption weight gain rate (*c_t_*) of the material and time (*t*) can be expressed by Fric′s law of diffusion as expressed by Equation (6):

(6) $$\frac{c_{t}}{c_{s}}=1-\frac{8}{\pi^{2}}\sum_{n=0}^{\infty }\frac{1}{{\left(2n+1\right)}^{2}}\exp\left[\frac{-D{\left(2n+1\right)}^{2}\pi^{2}t}{d^{2}}\right]$$

where *c_s_* is the saturated water absorption weight gain rate, *d* is the sample thickness, and *D* is the diffusion coefficient. In the initial stage of the water absorption process, Equation (6) can be approximated as Equation (7) as follows:

(7) $$\frac{c_{t}}{c_{s}}=\frac{4}{\pi^{1/2}}{\left(\frac{Dt}{d^{2}}\right)}^{1/2}$$

On this basis, another *K* = *c_s_*[16*Dt*/(π*d*^2^)]^1/2^, that is, Equation (7) can be approximated as Equation (8):

(8) $$c_{t}=Kt^{1/2}$$

In Equation (8), *K* is the water absorption rate of the material. The *c_s_* of the No. 5 sample is 1.23%, and the water absorption rate (*K*) obtained by using Equation (8) is 0.177 h^−1/2^. There is a free volume between the polymer molecules formed by the curing of the epoxy resin, which provides sufficient space for water molecules to diffuse and penetrate between molecules; and the epoxy resin has a large number of polar groups after curing. The opened hydroxyl group is a hydrophilic group; at the same time, these hydrophilic groups may form hydrogen bonds with water molecules, thereby allowing the material to absorb water. However, for buoyancy materials, the lightweight filler HGM is an inorganic filler, and its water absorption is deficient.

**Figure 8:**
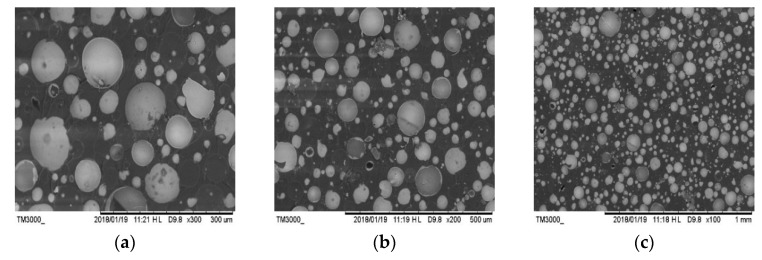
The cross-sectional morphology of AG-80 epoxy resin-based buoyancy material with 55% volume fraction of HGM. (**a**) The cross section of the No.5 sample is magnified 300 times. (**b**) The cross section of the No.5 sample is magnified 200 times. (**c**) The cross section of the No.5 sample is magnified 100 times.

The gap between HGM and epoxy resin matrix is the critical factor of increasing the water absorption rate and decreasing the compressive strength. SEM was used to observe the fracture surface of the No. 5 sample for buoyancy material. Through the observation of the microscopic morphology, the distribution of the HGM and the degree of fracture and the interface bonding between the epoxy resin matrix and HGM can be understood, thereby the influence factors of the properties of buoyancy material could be analyzed. Figure 8 shows the cross-sectional morphology of the No. 5 sample prepared by H32-type HGM with 55% *V*_H_. It can be seen from Figure 8 that HGM has different degrees of damage, but most of the HGM remains intact. Furthermore, Figure 8 exhibits the shape of the compression fracture surface, in which the compression process causes a large amount of HGM fracture. Moreover, it can be noted that the distribution of HGM is relatively uniform, and there is no more significant agglomeration. Besides, there is no clear separation and appearance of bubbles between the resin matrix and HGM interface, which is consistent with the results obtained by fitting the sample strength and HGM *V*_H_ curve.

**Figure 9:**
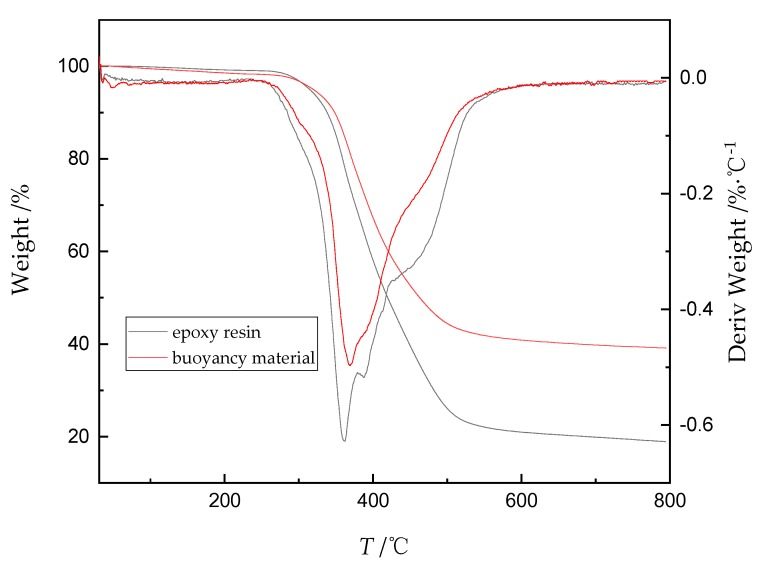
TGA thermographs of cured AG-80/m-XDA (*phr* = 26) and AG-80 epoxy resin-based buoyancy material (*V*_H_ = 55%) with heating rate of 10 °C/min in N_2_.

The TGA test is used to estimate the thermal stability of this type of buoyancy material. TGA data of cured AG-80/m-XDA (*phr* = 26) and AG-80 epoxy resin-based buoyancy material with 55% volume fraction of HGM were obtained at heating rate of 10 °C/min. Figure 9 exhibits the TGA thermographs for the weight and derivative-weight percentage (decomposition rate) against temperature for cured AG-80/m-XDA (*phr* = 26) and the No. 5 sample with the heating rate of 10 °C /min in N_2_. There are several decomposition peaks of derivative-weight percentage (DTG) curves in Figure 9, which indicates the thermal decomposition of these systems is a complex process. They remain thermally stable up to ~250 °C, and the AG-80 epoxy resin-based buoyancy material with HGM (*V*_H_ = 55%) starts to decompose at a slightly higher temperature. The high initial temperature may due to the HGM filler enhance the thermal stability of cured AG-80/m-XDA system [43]. The volume fraction of HGM is more than epoxy resin matrix, which blocks the decomposition of epoxy resin for a certain extent. Once the temperature rises above 260 °C, the epoxy resin matrix starts to decompose rapidly and the most lose with 260~600 °C. In this stage, the chain scission of the networks yields the combustible gases, water, amines, and gaseous aromatic compounds, etc. [44]. At the even high temperature, the decrease of the weight percentage becomes very slow, leaving about 19% and 39% residual substance at 800 °C, respectively. The main components of the residue are char and silica, respectively. Besides, the difference in weight percentage for two systems is basically consistent with the quality percentage of HGM added. The thermal decomposition process of two systems has less differences, which indicates that the decomposition of epoxy resin matrix is the main thermal decomposition reaction.

## 4. Conclusions

In this study, a novel HGM/AG-80 composite with low density, low water absorption, and high compressive strength was successfully prepared by employing m-XDA as a curing agent. AG-80 epoxy resin has excellent adhesion and compressive strength, and as a resin matrix for in-depth sea buoyancy materials, it can provide good compressive strength. HGM with hollow structure acts as the filler which can give low density and maintain compressive strength of AG-80 epoxy resin. With an increase in the curing degree, the *E*_a_ of the curing reaction gradually decreases. For the process of buoyancy material, the mass ratio of AG-80 with m-XDA is 100/26, and the curing process is pre-curing at 75 °C for 2 h, curing at 90 °C for 2 h, and post-curing at 100 °C for 2 h. For the buoyancy material, with the *V*_H_ of HGM increasing from 40% to 55%, the bulk density, compressive strength, and *E*/*ρ* of the samples ranged from 0.844 to 0.729 g/cm^3^, 120.69 to 108.78 MPa, and 143.00 to 149.22, respectively. The density and compressive strength decrease with the increase of HGM, but are gradually increasing. When the *V*_H_ of HGM was 55%, the water absorption of the composite is 1.23% and this type of buoyancy material can resist the temperatures of 250 °C.

## Figures and Tables

**Table 1:** The exothermic values for AG-80/m-XDA curing system with different mass ratios (*phr* = 20, 22, 24, 26, 28, 30) at a rate of 2.5 K/min.

Curing System	*phr*	*Q*/J·g^−1^
AG-80/m-XDA	20	544.7
	22	534.3
	24	512.5
	26	1383.1
	28	732.6
	30	525.5

**Table 2:** The characteristic temperatures and reaction enthalpy of AG-80/m-XDA curing system (*phr* = 26) obtained from DSC curves with different heating rates (*β* = 2.5 K/min, 5 K/min, 10 K/min, 15 K/min, and 20 K/min).

*β*/K·min^−1^	*T*_i_/°C	*T*_p_/°C	*T*_f_/°C	△*H*/J·g^−1^
2.5	76.9	93.7	100.8	−450.2
5	82.7	102.4	112.5	−432.9
10	89.2	113.9	128.7	−423.8
15	96.8	122.4	139.3	−473.5
20	104.6	129.1	147.4	−484.1

**Table 3:** The fitting results for AG-80/m-XDA curing system (*phr* = 26) obtained by FWO method at different conversion rates (*α* = 0.02~0.98).

*α*	Slope	Intercept	*r* ^2^	*E_a_*/kJ·mol^−1^
0.02	−5147.41	15.58	0.9316	93.71
0.05	−4506.62	13.51	0.9585	82.04
0.10	−4142.63	12.30	0.9682	75.41
0.15	−3975.30	11.72	0.9730	72.37
0.20	−3877.87	11.37	0.9766	70.59
0.25	−3803.00	11.10	0.9794	69.23
0.30	−3745.06	10.89	0.9810	68.18
0.35	−3705.35	10.74	0.9830	67.45
0.40	−3663.82	10.59	0.9877	66.70
0.45	−3624.80	10.45	0.9861	65.99
0.50	−3590.99	10.33	0.9870	65.37
0.55	−3569.71	10.24	0.9890	64.98
0.60	−3522.66	10.09	0.9892	64.13
0.65	−3484.29	9.96	0.9899	63.43
0.70	−3443.85	9.82	0.9907	62.69
0.75	−3399.63	9.68	0.9913	61.89
0.80	−3343.24	9.50	0.9918	60.86
0.85	−3275.40	9.29	0.9920	59.63
0.90	−3195.53	9.05	0.9923	58.17
0.95	−3093.60	8.73	0.9922	56.32
0.98	−2999.04	8.42	0.9921	54.60

**Table 4:** Extrapolation values of the characteristic temperatures for AG-80/m-XDA curing system (*phr* = 26) with different heating rates.

Extrapolated Temperatures	*T*/°C	*r* ^2^
*T*_i_	73.92	0.997
*T*_p_	91.43	0.987
*T*_f_	98.20	0.983

**Table 5:** The comprehensive properties of cured AG-80/m-XDA system (*phr* = 26) and AG-80 epoxy resin-based buoyancy materials with different volume fraction (*V*_H_ = 40%, 45%, 50%, and 55%) of hollow glass microspheres (HGM).

Sample *No.*	*V*_H_/%	*ρ*_CAL_/g·cm^−3^	*ρ*/g·cm^−3^	*E*_CAL_/MPa	*E*/MPa	*E/ρ*
1	0	——	1.208	——	126.14	104.42
2	40	0.849	0.844	115.98	120.69	143.00
3	45	0.804	0.812	115.10	116.65	143.66
4	50	0.759	0.764	113.67	112.07	146.69
5	55	0.714	0.729	111.48	108.78	149.22
